# dl‐3‐n‐butylphthalide preserves white matter integrity and alleviates cognitive impairment in mice with chronic cerebral hypoperfusion

**DOI:** 10.1111/cns.13189

**Published:** 2019-07-23

**Authors:** Qin‐Yu Han, He Zhang, Xi Zhang, Dong‐Sheng He, Sun‐Wei Wang, Xiang Cao, Yu‐Tian Dai, Yun Xu, Li‐Juan Han

**Affiliations:** ^1^ Department of Neurology Drum Tower Hospital, Medical School of Nanjing University Nanjing China; ^2^ Jiangsu Key Laboratory for Molecular Medicine Medical School of Nanjing University Nanjing China; ^3^ Department of Neurology The Northern Area of Suzhou Municipal Hospital, Nanjing Medical University Suzhou China; ^4^ Department of Neurology Wuxi People's Hospital, Nanjing Medical University Wuxi China

**Keywords:** chronic cerebral hypoperfusion, dl‐3‐n‐butylphthalide, vascular cognitive impairment, white matter lesions

## Abstract

**Aims:**

Effects of dl‐3‐n‐butylphthalide (NBP) on white matter damage and cognitive impairment in vascular cognitive impairment (VCI) have not been well studied. This study aimed to investigate the effects of NBP treatment on chronic cerebral hypoperfusion‐induced white matter lesions and cognitive dysfunction in mice.

**Methods:**

Mice were subjected to bilateral common carotid artery stenosis (BCAS) for over 30 days. The cerebral blood flow was detected using a laser Doppler flowmetry. Cognitive functions were assessed by several behavioral tests. We also evaluated the effects of NBP on the blood‐brain barrier (BBB) disruption and reactive astrogliosis, using Evans Blue extravasation, Western blot, CBA, and immunofluorescence in BCAS mice and cultured astrocytes.

**Results:**

The results indicated that NBP treatment attenuated spatial memory dysfunction while promoted cerebral perfusion and white matter integrity in BCAS mice. Moreover, NBP treatment prevented BBB leakage and damage of endothelial cells, as well as disruption of endothelial tight junctions. Furthermore, NBP administration effectively decreased the number of activated astrocytes and pro‐inflammatory cytokines, as well as the production of MMPs, in BCAS‐induced mice and LPS‐stimulated astrocytes.

**Conclusion:**

Our results indicated that NBP represents a promising therapy for chronic cerebral hypoperfusion‐induced white matter damage and cognitive impairment.

## 1 INTRODUCTION

**Figure 1:**
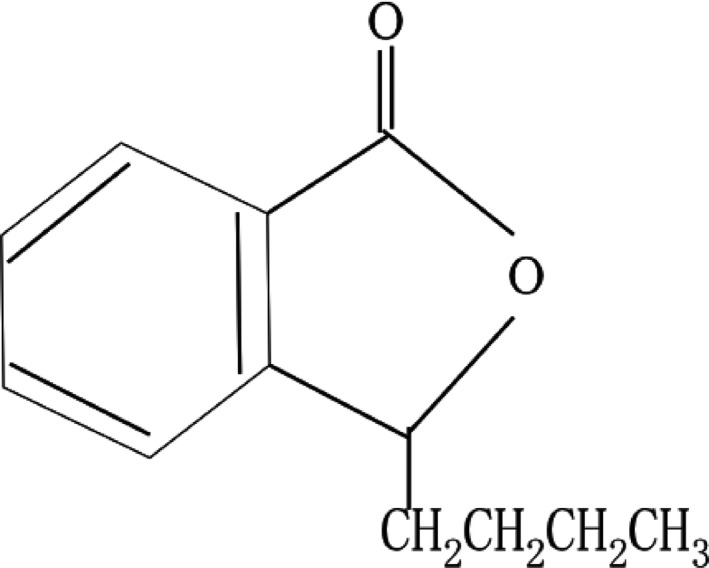
The chemical structure of dl‐3‐n‐butylphthalide (NBP)

Vascular cognitive impairment (VCI) is termed as a spectrum of cognitive dysfunction associated with vascular disease origins from mild cognitive impairment to dementia.^1^, ^2^ White matter damage due to chronic cerebral hypoperfusion is involved in a variety of neurological disorders including VCI.^1^, ^3^ White matter hyperintensities (WMH) are the main manifestation of white matter damage, while cognitive impairment and subjective impairment are associated with severe WMH.^4^, ^5^ White matter lesions in VCI are characterized by myelin sheath loss, inflammation, and increased BBB permeability.^6^, ^7^, ^8^ Activation of glial cells and excessive production of inflammatory cytokines after hypoxia, including TNFα and IL‐6, results in decreased blood flow, damage to white matter, and cognitive dysfunction.^9^, ^10^, ^11^ Using dynamic contrast‐enhanced magnetic resonance imaging, BBB leakage in the white matter has been demonstrated in patients with VCI.^12^ Currently, there are no known effective treatments for VCI other than modifying vascular risk factors. Intriguingly, therapeutic approaches aiming to reduce white matter damage have been demonstrated to effectively prevent cognitive decline in animal models of VCI.^13^, ^14^

dl‐3‐n‐butylphthalide (Figure 1) is derived from l‐3‐n‐butylphthalide which is initially extracted from seeds of Chinese celery, *Aplumgraveolens*. dl‐3‐n‐butylphthalide was approved by the State Food and Drug Administration of China for treatment for patients with acute ischemic stroke in 2002.^15^ Studies have demonstrated that NBP exerts multi‐target protective effects against ischemic stroke, including reducing oxidative stress, inhibiting inflammatory responses, alleviating neuronal apoptosis, ameliorating BBB damage, and increasing regional blood flow.^16^, ^17^, ^18^ Moreover, emerging evidence indicates that NBP also have beneficial effects on cognitive function of Alzheimer's Disease (AD) and vascular dementia,^17^, ^19^, ^20^ through regulating endoplasmic stress, reducing neuronal apoptosis, and glial reactivity.^21^, ^22^, ^23^

Yet, limited data are available about the effects of NBP on chronic cerebral hypoperfusion‐induced white matter damage and cognitive impairment. As the most valid animal model for VCI,^9^ multiple pathophysiological manifestations of VCI have been demonstrated in BCAS mice, including reduced cerebral blood flow (CBF), myelin damage, death of oligodendrocytes, neuroinflammation, and BBB breakdown.^24^ Therefore, in the present study, we aimed to investigate the effects of NBP on white matter damage and cognitive dysfunction in BCAS mice model of VCI.

## 2 METHODS

### 2.1 Animals and model of chronic cerebral hypoperfusion

Male C57BL/6J mice (8‐10 weeks, weight 25‐28 g) were provided by the Animal Center of Nanjing Drum Tower Hospital. They were housed in a specific pathogen‐free (SPF) environment on a 12‐hour light/12‐hour dark cycle, allowed free access to water and standard food. Chronic cerebral hypoperfusion was induced by BCAS as previously reported.^9^ Briefly, mice were anaesthetized with 4% chloral hydrate (10 mL/kg) by intraperitoneal injection. Both common carotid arteries (CCAs) were exposed through a midline cervical incision. The right CCA was gently lifted and placed between the loops of a microcoil (Inner diameter 0.18 mm, pitch 0.50 mm, total length 2.5 mm, purchased from Sawane Spring Co.) below the carotid bifurcation. The microcoil was twined by rotating it around the CCA. The same procedure was conducted to the left CCA. Sham‐operated mice underwent the same surgical procedure without microcoil implantation. The mortality rate was about 10%. All experimental procedures and animal care were conducted according to the Standard Medical Laboratory Animals Care and Use Protocols (Ministry of Health PR China, 1998) and the Experimental Animal Administration Committee of Nanjing University.

### 2.2 Cerebral blood flow measurements

The CBF was measured in mice using a laser Doppler flowmetry (LDF).^25^ The LDF probe was placed perpendicularly to the skull at 1 mm posterior and 2.5 mm lateral to the bregma by a fixed plastic guide cannula (3 mm in outer diameter, 2 mm in inner diameter, 4 mm in length) using dental resin. Cerebral blood flow was recorded before BCAS, immediately after BCAS, at day 30 and day 60 after BCAS with or without NBP treatment. Perfusion images were acquired using PSI system with a 70‐mW built‐in laser diode for illumination and a 1388 × 1038 pixels CCD camera mounted 10 cm above the skull (speed 19 Hz, exposure time 6 ms). Cerebral blood flow values recorded before surgery were set as baseline (100%). The relative CBF was determined as the percentage value normalized to the presurgical baseline for each animal.

### 2.3 Chemicals and experimental groups

dl‐3‐n‐butylphthalide (formula: C12H14O2; 99.6% purity; Shijiazhuang Pharmaceutical Group NBP Pharmaceutical Co., Ltd.) was extracted from celery seed. The extracted liquid was yellow and oil‐like, which was directly diluted with corn oil. Thirty BCAS mice were randomized into 3 groups (n = 10): the NBP‐treated group (BCAS + NBP), the vehicle‐treated group (BCAS + corn oil) and the untreated group (BCAS only). Ten sham‐operated mice (sham group) were assigned to the control group. The mice in the BCAS + NBP group were treated with 80 mg/kg NBP by gavage. All mice were treated once a day for 60 days.

### 2.4 Morris water maze test

Neurological deficit scores and Morris water maze were most commonly used in stroke and VCI, respectively.^26^ As previously described,^27^ spatial learning and memory was evaluated by the Morris water maze both at baseline and after BCAS. During the acquisition phase (days 1‐5), the mice were trained to find the platform within 60 seconds. They were allowed to stay on the platform for 30 seconds to get familiar with their surroundings in case that they could not find it within 60 seconds, and 60 seconds was the latency. In the probe test (day 6), the platform was removed and the mice were allowed to swim freely for 60 seconds, and the number of platform crossing, the time in the target quadrant and the latency to the target quadrant were recorded. All data were analyzed using the ANY‐maze system (Stoelting).

### 2.5 Y‐maze test

Y‐maze test was performed at days 7, 14, 21, 30, and 60 after BCAS as previously reported.^10^ Mice were placed at the intersection of the three arms (marked as A, B, C) with equal length and angle, and allowed to move freely through the maze for 8‐minute sessions. Spontaneous alterations were defined as the consecutive entry into all three different arms to form a triplet of non‐repeated components, with the following six cases: ABC, ACB, CAB, BCA, CBA, and BAC. The percentage of spontaneous alterations (%) was defined as the number of spontaneous alterations in behavior/(the total number of arm entries − 2) × 100.

### 2.6 Novel object recognition test

The novel object recognition test was performed to detect recognition memory as previously described.^10^ During the adaptation session, mice were objected to explore the experimental box freely for 15 minutes. In the test, mice were allowed to explore the box with two identical red cuboid objects for 10 minutes. One hour later, one of the objects was replaced by a green cube object. Mice were placed in the box again to explore for 5 minutes. Exploration was defined as sniffing or touching the stimulus object with the nose or forepaws. The total time spent exploring each object was measured by an operator who was blinded to the group assignments. The percentage of exploratory preference was defined as time of exploration spent with the novel object/the total time of exploration spent with both objects.

### 2.7 Modified neurological severity score

Motor functional outcome was evaluated through modified neurological severity score (mNSS).^28^ Mice were evaluated by raising by tail, placing on floor, and beam balance walking, and then, all test scores were added into the mNSS score. The mNSS evaluations were performed every 10 days after BCAS.

### 2.8 Rotarod performance test

Before formal testing, training was conducted five times at a speed on 20/30/40 rpm for 5 minutes. In the fatigue analysis, mice were placed on the turning device (YLS‐4C) with 40 rpm speed, and the time when mice fell off was recorded. If the mouse did not fall within 5 minutes, the recording time was five minutes.^29^

### 2.9 Evans blue extravasation and qualification

Mice were sacrificed at day 60 after BCAS to evaluate BBB dysfunction. Six hours after intraperitoneal injection of 1 mL of 4% Evans blue (EB, Sigma Aldrich, Inc), mice were anesthetized and then perfused transcardially with 40 mL of phosphate‐buffered saline (PBS). The brain tissues were then placed in N, N‐dimethylformamide, rapidly homogenized with a homogenizer and centrifuged at 25 000 rcf for 45 minutes. The supernatant was taken and measured with a microplate reader for the absorbance at 620 nm. Evans blue levels were calculated according to the standard curve.

### 2.10 Immunofluorescence staining and quantification

The mice were sacrificed by cardiac perfusion with 0.9% saline, and their brains were cut into 20 μm coronal sections after being fixed in 4% paraformaldehyde. The brain sections were incubated with anti‐MBP (1:400, Abcam), anti‐SMI32 (1:400, Covance), anti‐CD31 (1:200, Santacruz), anti‐claudin‐5 (1:400, Abcam), anti‐occludin (1:400, Invitrogen), anti‐GFAP (1:400, CST), anti‐MPO (1:400, CST), anti‐MMP‐9 (1:400, CST) antibodies, respectively, overnight at 4°C. After being washed with PBS, the sections were incubated with the appropriate secondary antibodies for 2 h in the dark at room temperature. The sections were imaged using an Olympus microscope with an IX71 digital camera (Olympus), and the images were quantified with IPP 6.0 software. All evaluations were performed by a blinded investigator.

### 2.11 Cell isolation, culture, and treatment

Primary astrocytes were isolated from the brains of newborn C57BL/6J mice (1‐3 days) according to a previous report.^30^ After 2 weeks, primary astrocytes were plated in 6‐well culture plates at a density of 1 × 10^6^ cells/well. After overnight incubation, the cells were stimulated by 100 ng/mL LPS for 3 hours with or without treatment of 1 μM NBP.

### 2.12 Cytometric bead array

The supernatant from wells containing only astrocytes (vehicle), wells containing LPS (LPS) and wells containing NBP (LPS + NBP) were collected and measured using a CBA Mouse Inflammation Kit from BD Biosciences containing IL‐6, IL‐10, MCP‐1, TNFα, IFN‐ɣ, and IL‐12p7. Standard curves were reconstituted according to the manufacturer's instructions. The samples were used neat, and the assay was performed in Falcon 12 × 75‐mm sample acquisition tubes (Corning Incorporated). Mixed capture beads (50 μL), each unknown sample (50 μL) and PE detection reagent (50 μL) were added to all assay tubes and incubated for 2 hours at room temperature, protected from light. The beads were washed with Washing Buffer (1 mL) and centrifuged at 200 *g* for 5 minutes. After resuspended in 300 μL of Washing Buffer, the beads were analyzed on FCAP Array software (Soft Flow Hungary Ltd.).

### 2.13 Western blot assay

Mice were euthanized and decapitated, and their striatum was quickly dissected on ice. Brain samples and treated astrocytes in vitro were homogenized in RIPA buffer (Beyotime Biotechnology) containing a mixture of phosphatase and protease inhibitors. The protein concentration was then determined using a BCA kit (Beyotime Biotechnology). Western bolt was executed according to a standard protocol. The hydrophilic polyvinylidene fluoride (PVDF) membrane, which was blocked in 5% skim milk powder for 1 hour at room temperature, was incubated in anti‐GAPDH (1:5000, Bioworld), anti‐claudin‐5 (1:500, Abcam), anti‐occludin (1:500, Invitrogen), anti‐CD31 (1:500, Abcam), anti‐MPO (1:500, CST), anti‐MMP‐2 (1:500, CST) and anti‐MMP‐9 (1:500, CST) antibodies overnight at 4°C. After washing with TBS buffer, the membrane was incubated with the appropriate HRP‐conjugated secondary antibody for 2 hours at room temperature. The immunoreactive bands were visualized with a chemiluminescent reagent provided with an ECL kit (Bioworld). The blot strength was detected using ImageJ software.

### 2.14 Gelatin zymography

Proteins from isolated brain tissues were subjected to gelatin zymography to analyze MMP‐2/9 as previously described with minor modifications.^31^ Protein samples (20 μL) were loaded on Zymogram Plus Gels (Invitrogen). Gels were washed in Zymogram Renaturing Buffer (Invitrogen) for 30 minutes and then incubated for 24 h in Zymogram Developing Buffer (Invitrogen) at 37°C followed by staining with Coomassie blue. Gels were destained until clear bands of gelatinolysis appeared.

### 2.15 Statistical analysis

All results were presented as mean values ± standard error of the mean (SEM) and analyzed by SPSS 16.0 statistical analysis software (SPSS). Multiple comparisons were made using a two‐way ANOVA followed by the Bonferroni post hoc test. The Student *t* test was used for two‐group comparisons. Results were considered to be statistically significant at *P* ≤ 0.05.

## 3 RESULTS

### 3.1 NBP ameliorated cognitive impairment after BCAS

**Figure 2:**
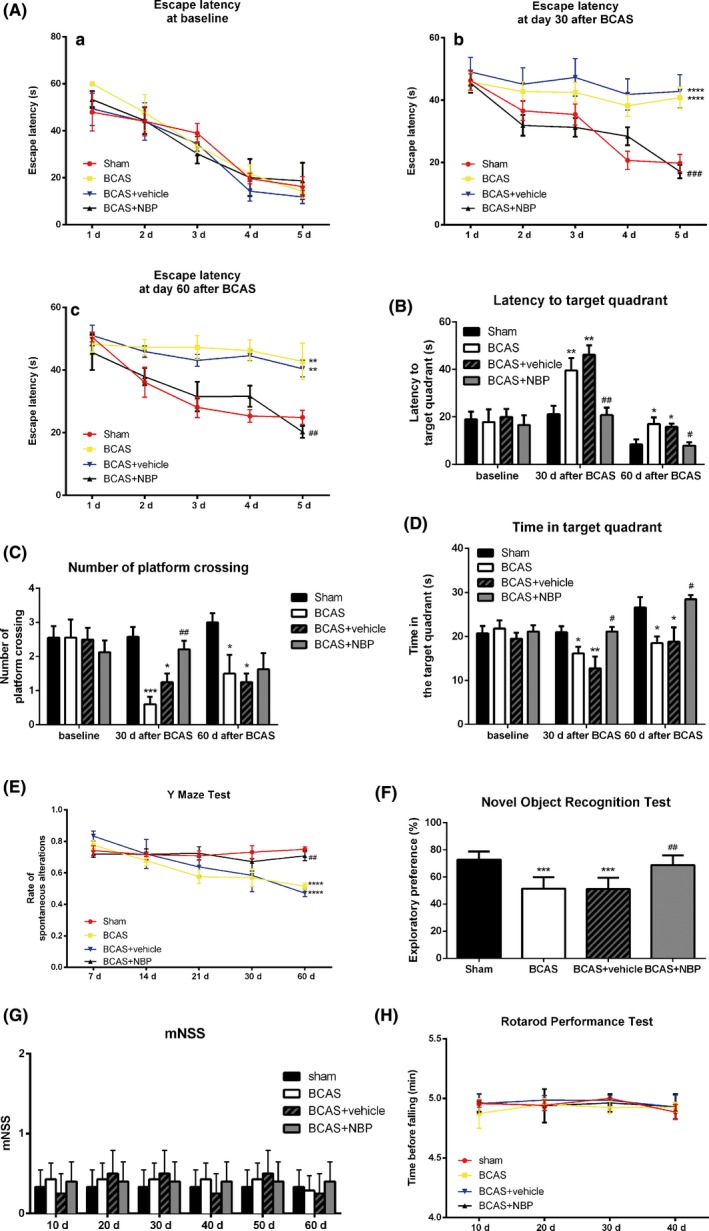
NBP ameliorated cognitive impairment after BCAS. A, The escape latency of mice during the acquisition phase of Morris water maze test at different time points. B, C, D, The latency to the target quadrant, the frequency of platform crossing and the time spent in target quadrant in the probe test of Morris water maze test at different time points. E, The percentage of spontaneous alterations in the Y‐maze test at different time points. F, The exploratory preference the novel object recognition test at day 60 after BCAS. G, The modified neurological severity score (mNSS) at different time points. H, The time before falling in the rotarod performance test at different time points. All data are presented as mean ± SEM (n = 10/group). *P < 0.05, **P < 0.01, ***P < 0.005, ****P < 0.001, compared with sham group; #*P* < 0.05, ##*P* < 0.01, ####*P* < 0.001, compared with BCAS group

To determine whether NBP treatment promoted cognitive impairment in mice after BCAS, behavioral tests were performed. Morris water maze test was used to evaluate mice spatial learning and memory. There were no significant difference in the escape latency among all groups before BCAS procedure (baseline) (Figure 2A a). However, at both day 30 and day 60 after BCAS, the BCAS mice spent more time searching the platform during the acquisition phase compared to the sham group, whereas NBP treatment reduced the latency (Figure 2A b,c). In the probe test, the BCAS mice had a prolonged latency to the target quadrant (Figure 2B), a decreased number of platform crossing (Figure 2C), and spent significantly less time in the target quadrant (Figure 2D). In contrast, mice treated with NBP had a better memory than BCAS group, as evidenced by a shorter latency to the target quadrant (Figure 2B), a significantly increased frequency of crossing the platform (Figure 2C), and spending less time in target quadrant in comparison with the BCAS group (Figure 2D).

At the same time, we performed Y‐maze test to evaluate the discriminative learning and working memory of mice. The results showed that at day 60 after BCAS, the BCAS mice had a lower percentage of spontaneous alterations than mice of the sham group. However, the percentage of spontaneous alterations after NBP treatment was significantly increased compared with the BCAS group (Figure 2E).

At day 60 after BCAS, mice were also subjected to the novel object recognition test. The results showed that the sham group was more inclined to be attracted by a novel object, which is a characteristic exploratory feature of rodents. Compared with the sham group, the BCAS group had less curiosity about the exploration of the new object, as can be seen from the significantly reduced exploratory preference. However, NBP treatment significantly restored exploratory preference compared to the BCAS group (Figure 2F).

We used mNSS score and rotarod performance test to evaluate motor function. No significant difference was found among the mNSS score (Figure 2G) and time before falling in the rotarod performance test (Figure 2H) of all groups, indicating that cognitive impairment in mice after BCAS was not affected by motor function with or without NBP treatment. Thus, these data from the behavioral tests demonstrated that NBP treatment ameliorated cognitive deficits in learning and memory functioning in mice subjected to chronic cerebral ischemia.

### 3.2 NBP preserved white matter integrity after BCAS

**Figure 3:**
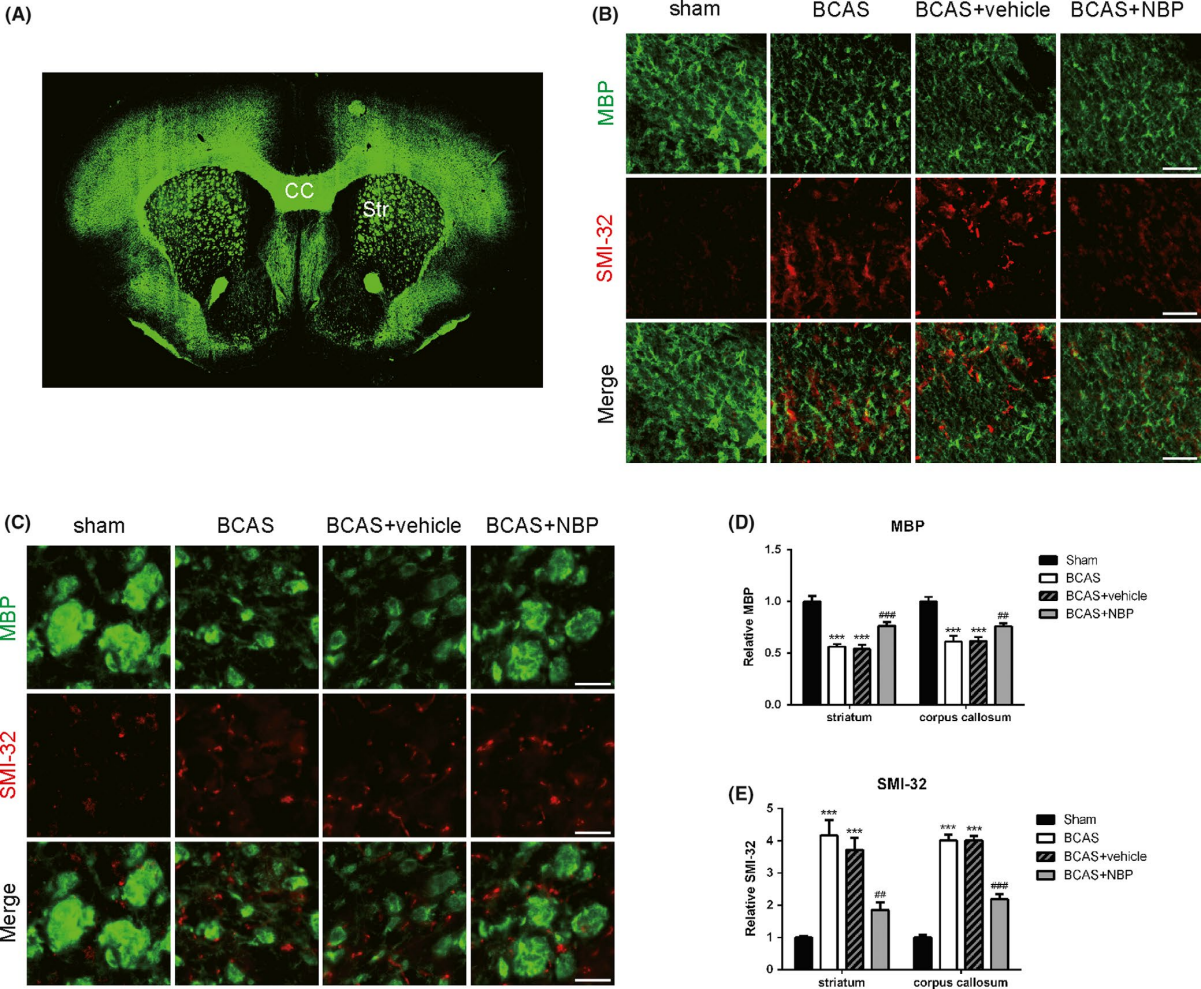
NBP preserved white matter integrity after BCAS. A, Schematic diagram showing coronal section of the mouse brain at the level of 0.5‐1 mm anterior to bregma. CC = corpus callosum, Str = striatum. B, C, Representative images of myelin basic protein (MBP; green) and SMI‐32 (red) immunostaining in the corpus callosum and striatum at day 30 after BCAS. Scale bar: 50 μm. D, Quantification of immunofluorescent intensity of MBP area (n = 4/group). E, Quantification of immunofluorescent intensity of SMI‐32 area (n = 4/group). All data are presented as mean ± SEM. ***P < 0.005, compared with sham group; ##*P* < 0.01, ###*P* < 0.005, compared with BCAS group

White matter damage is an important manifestation of chronic ischemic brain injury. Therefore, we used immunofluorescence, labeling MBP, a marker of myelin integrity, and SMI32, a marker of axonal injury, to evaluate the white matter integrity after BCAS. Myelin loss (Figure 3A) in company of axonal damage was detected in the striatum (Figure 3B) and corpus callosum (Figure 3C) of the BCAS group brains at day 30 after the surgery. dl‐3‐n‐butylphthalide treatment significantly preserved white matter integrity after BCAS, as measured by the increased MBP staining intensity (Figure 3D) and decreased SMI32 staining intensity (Figure 3E) relative to the BCAS group. These results indicated that NBP treatment reduced white matter damage in BCAS‐induced mice.

### 3.3 NBP reduced damage of endothelial tight junctions after BCAS

**Figure 4:**
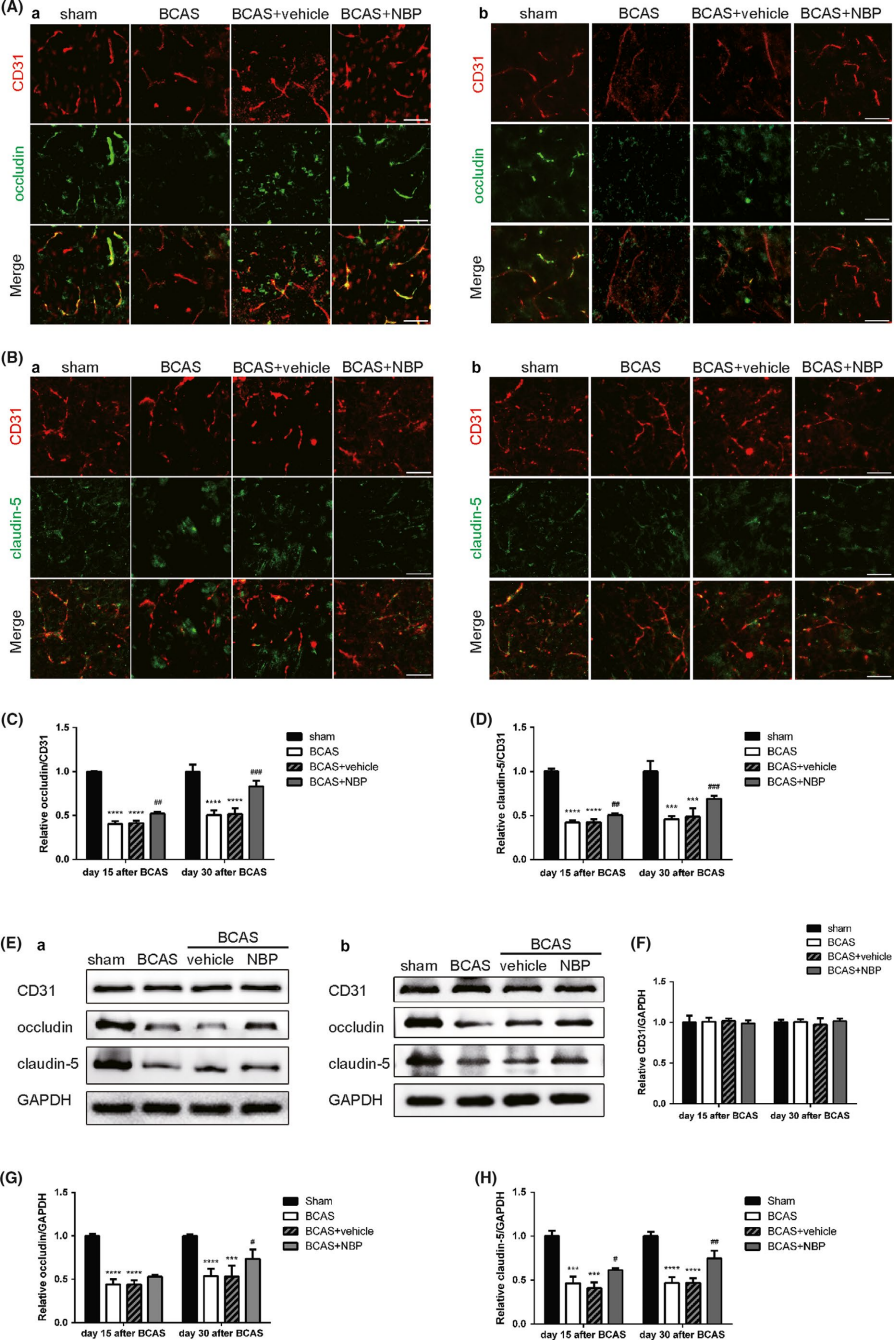
NBP reduced damage of endothelial tight junctions after BCAS. A, Immunofluorescent images of occludin (green)/CD31 (red) colocalization at day 15 (a) and day 30 (b) after BCAS. Scale bar: 50 μm. B, Immunofluorescent images of claudin‐5 (green)/CD31 (red) colocalization at day 15 (a) and day 30 (b) after BCAS. Scale bar: 50 μm. C, Quantification of immunofluorescent intensity of occludin to CD31 area (n = 4/group). D, Quantification of immunofluorescent intensity of claudin‐5 to CD31 area (n = 4/group). E, Representative immunoblots probed with antibodies against CD31, occludin, claudin‐5 and GAPDH at day 15 (a) and day 30 (b) after BCAS. F, Quantification of the levels of CD31 normalized to GAPDH (n = 4). G, Quantification of the levels of occludin normalized to GAPDH (n = 4). H, Quantification of the levels of claudin‐5 normalized to GAPDH (n = 4). All data are presented as mean ± SEM. ***P < 0.005, ****P < 0.001, compared with sham group; #*P* < 0.05, ##*P* < 0.01, ###*P* < 0.005, compared with BCAS group

The permeability of BBB has been found to be increased significantly 2 weeks after moderate and sustained cerebral ischemia. Cerebral endothelial tight junction proteins, such as occludin and claudin‐5, play a critical role in maintenance of the BBB. To investigate whether endothelial tight junctions were damaged after BCAS, we used immunofluorescence, labeling occludin (Figure 4A), and claudin‐5 (Figure 4B), both endothelial tight junction proteins, and CD31, a marker of endothelial cells. The number of occludin‐immunoreactive and claudin‐5‐immunoreactive endothelial cells was decreased 15 days after the BCAS surgery. Similar results were found at day 30 after BCAS. Treatment with NBP reduced damage of endothelial tight junctions, as measured by the increased ratio of occludin/claudin‐5 to CD31 staining intensity relative to the BCAS group (Figure 4C,4). On the other hand, we evaluated the damage of endothelial cells using Western blot assay (Figure 4E). Similar to the results of immunofluorescence staining, the expression of occludin and claudin‐5 both decreased after BCAS, while the treatment of NBP increased the expression of these two proteins compared to the BCAS group (Figure 4G,H). However, there was no obvious difference in the expression of CD31 among all groups (Figure 4F). To conclude, NBP treatment reduced damage of endothelial tight junctions in mice after BCAS.

### 3.4 NBP suppressed astrocyte activation and MMPs levels after BCAS

**Figure 5:**
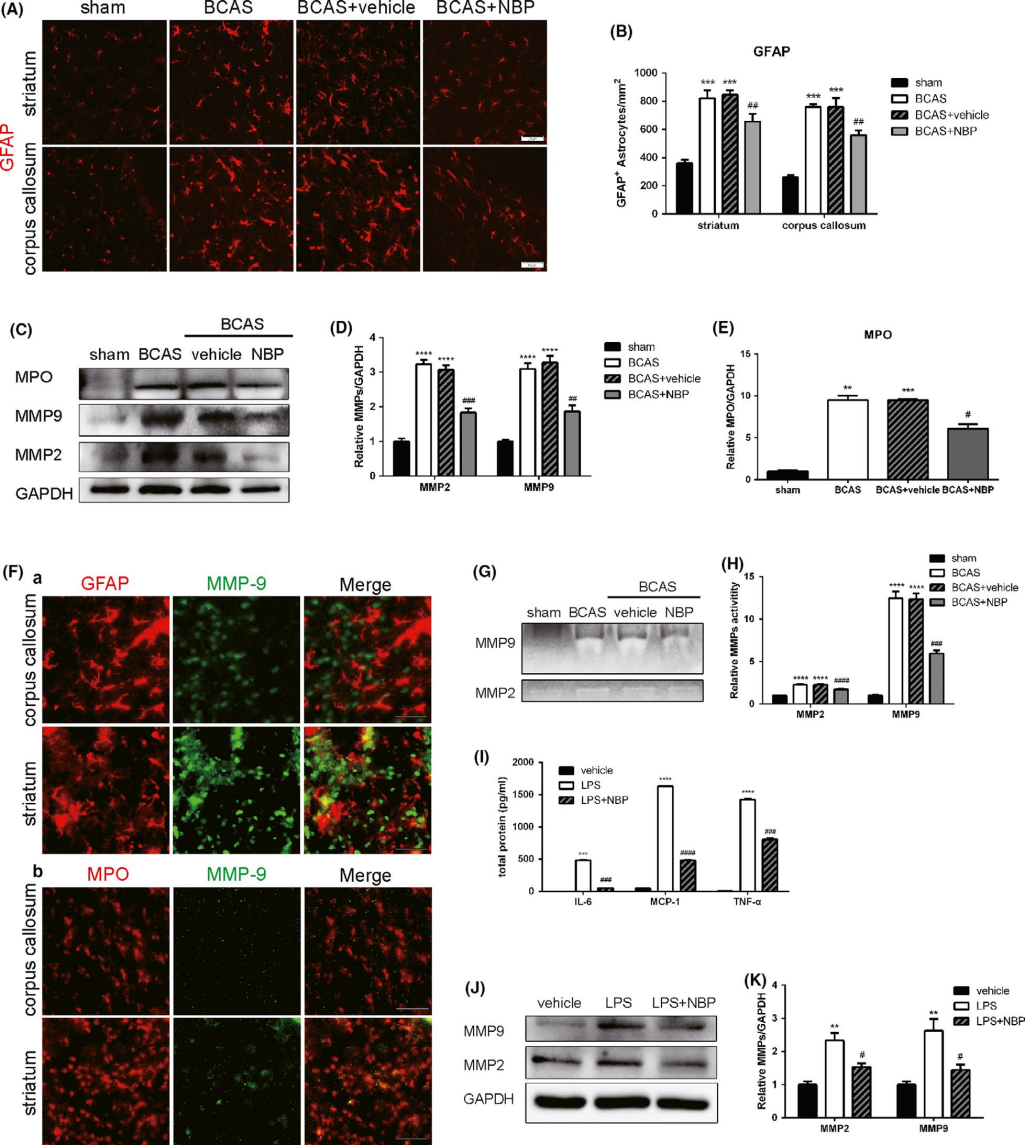
NBP suppressed astrocyte activation and MMPs activity after BCAS. A, Representative images of GFAP (red) immunostaining in the striatum and corpus callosum at day 15 after BCAS. Scale bar: 50 μm. B, Quantification of immunofluorescent number of GFAP‐positive cells (n = 4/group). C, Representative immunoblots probed with antibodies against MPO, MMP‐9, MMP‐2 and GAPDH at day 15 after BCAS. D, Quantification of the levels of MMP‐9 and MMP‐2 normalized to GAPDH (n = 4). E, Quantification of the levels of MPO normalized to GAPDH (n = 4). F, Representative images of GFAP (red, a) or MPO (red, b) and MMP‐9 (green) immunostaining in the corpus callosum and striatum at day 15 after BCAS. Scale bar: 50 μm. G, Results of gelatinase zymography for activities of MMP‐9 and MMP‐2 at day 15 after BCAS. H, Statistical results for MMP‐9 and MMP‐2 activity respectively. All data are presented as mean ± SEM. **P < 0.01, ***P < 0.005, ****P < 0.001, compared with sham group; #*P* < 0.05, ##*P* < 0.01, ###*P* < 0.005, ####*P* < 0.001, compared with BCAS group. I, Graph showing the levels of secreted TNF‐α, IL‐6 and MCP‐1 in LPS‐stimulated astrocyte supernatants measured using CBA (n = 4). J, Representative immunoblots probed with antibodies against MMP‐9, MMP‐2 and GAPDH. K, Quantification of the levels of MMP‐9 and MMP‐2 normalized to GAPDH (n = 4). All data are presented as mean ± SEM. **P < 0.01, ***P < 0.005, ****P < 0.001, compared with vehicle group; #*P* < 0.05, ###*P* < 0.005, ####*P* < 0.001, compared with LPS group

It has been reported that white matter changes are mediated by inflammation and glial activation. We evaluated the effects of NBP treatment on astrocyte activation in the striatum and corpus callosum of BCAS mice using immunofluorescence (Figure 5A). The results demonstrated a stronger presence of GFAP‐positive astrocytes within mice in the BCAS group, whereas NBP treatment reduced the number of activated astrocytes (Figure 5B). Meanwhile, we measured MMPs levels using Western blot assays (Figure 5C). The results showed that the expression of MMP‐9 and MMP‐2 both increased after BCAS, which was significantly reduced by treatment of NBP (Figure 5D). The results of Western blot revealed that BCAS increased the expression of myeloperoxidase (MPO), a marker of neutrophil, whereas NBP decreased the expression of MPO in the BCAS brains (Figure 5G).Moreover, we used immunofluorescence, labeling GFAP or MPO and MMP‐9, to test which cell is the main cell type releasing MMP‐9 after BCAS. The results showed that activated microglia released more MMPs than neutrophils.

At the same time, gelatin zymography was used to detect MMPs activity (Figure 5G). BCAS procedure remarkably increased activities of MMP‐2, MMP‐9, whereas NBP treatment inhibited the BCAS‐induced activities of MMP‐2, MMP‐9 in the brain (Figure 5H).

Furthermore, we investigated whether NBP reduced the levels of pro‐inflammatory cytokines in LPS‐stimulated astrocyte supernatants in vitro. CBA results showed that the levels of TNF‐α and IL‐6 were remarkably increased in LPS‐stimulated astrocyte supernatants, and NBP treatment significantly reduced the levels of TNF‐α and IL‐6 (Figure 5I). We also measured MMPs levels of LPS‐stimulated astrocytes using Western blot assays (Figure 5J). The expression of MMP‐9 and MMP‐2 both increased after LPS stimulation, which was significant decreased by NBP treatment (Figure 5K).

Overall, these data demonstrated that NBP administration attenuated astrocyte activation and neutrophil infiltration and reduced both MMPs levels and activity after BCAS in mice. It also reduced the pro‐inflammatory factors and MMPs released by astrocytes after LPS stimulation.

### 3.5 NBP promoted BBB integrity and cerebral perfusion after BCAS

**Figure 6:**
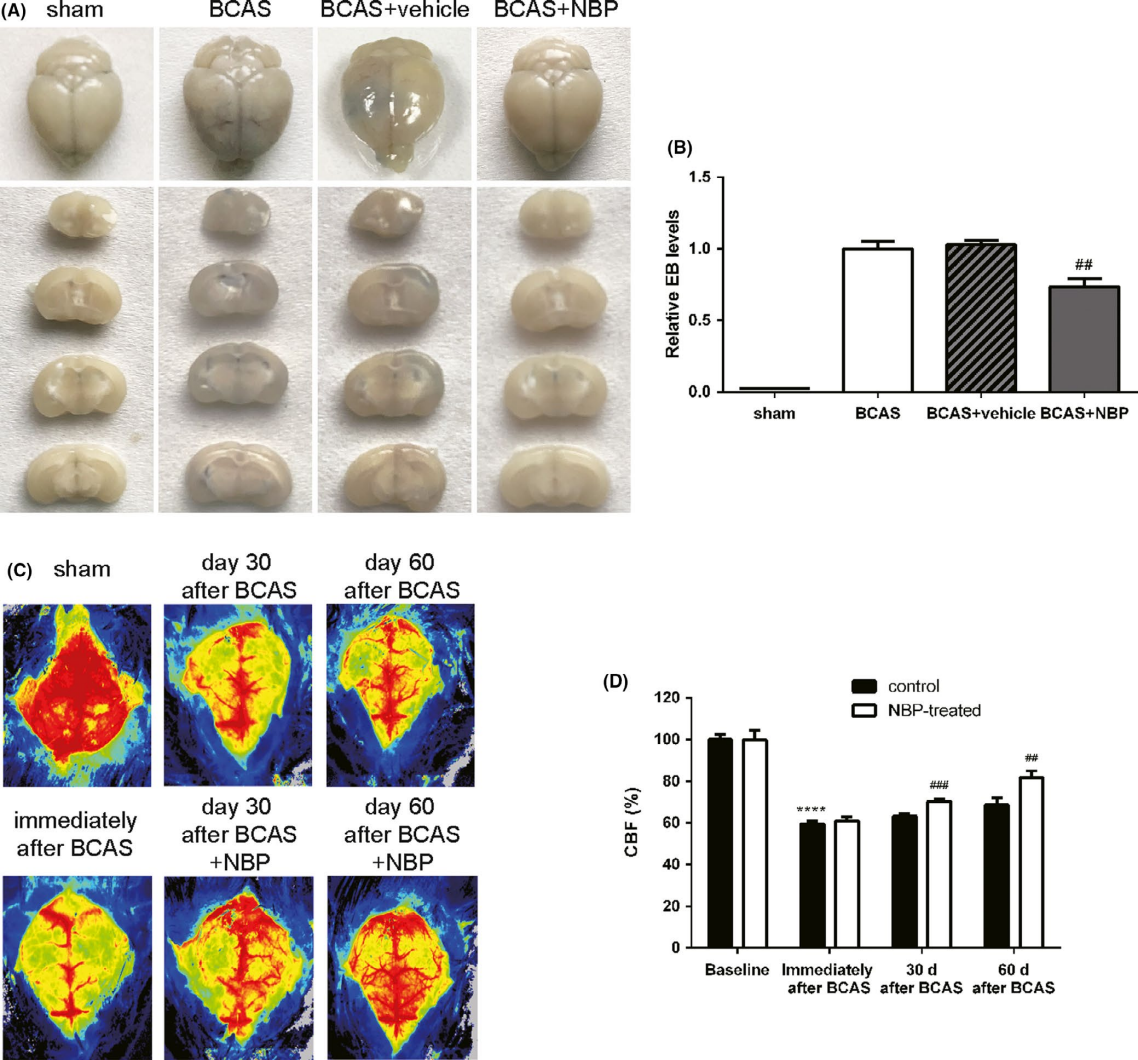
NBP promoted BBB integrity and cerebral perfusion after BCAS. A, Representative images of Evans blue extravasation at day 30 after BCAS. B, Quantitative analysis of Evans Blue extravasation. C, Representative images of cerebral perfusion at different time points. D, Quantitative analysis of CBF measurement. All data are presented as mean ± SEM. ****P < 0.001, compared with sham group; ##*P* < 0.01, ###*P* < 0.005, compared with BCAS group

Several lines of evidence indicate that the activation of glial cells and MMPs leads to BBB disruption in the course of the disease associated with white matter damage, and we therefore evaluated the BBB integrity using Evans blue extravasation test. The results showed that the leakage of Evans blue was increased in BCAS group, which was relatively reduced by the treatment of NBP (Figure 6A, B). Consistently, cerebral perfusion detected by CBF showed a significantly decrease in BCAS mice compared with sham‐operated mice, suggesting the successful induction of global hypoperfusion in BCAS mice. A slight spontaneous improvement in the CBF was detected in all BCAS groups with time and over days. However, in comparison to untreated BCAS group, there was a significant increase in CBF in mice treated with NBP at day 30 and day 60 after BCAS, respectively (Figure 6C, D). Our results demonstrated that NBP promoted BBB integrity and cerebral perfusion after BCAS surgery.

## 4 DISCUSSION

Chronic cerebral hypoperfusion induced by bilateral common carotid artery stenosis (BCAS) with microcoils reliably reproduces both clinical and pathological features of VCI.^9^, ^32^ In the present study, using this well‐established mouse model of VCI, we demonstrated that chronic cerebral hypoperfusion induced by BCAS caused cognitive deficits, CBF decline as well as white matter damage in mice subcortical area. Intriguingly, these changes were successfully attenuated by treatment with NBP, along with inhibition of astrogliosis and preservation of BBB integrity in BCAS mice. These results suggested that NBP exerts multiple beneficial effects on BCAS‐induced cognitive impairment.

Blood‐brain barrier breakdown which is often associated with cerebral microangiopathies plays critical roles in chronic cerebral hypoperfusion‐induced white matter changes and cognitive decline,^1^, ^33^ which has been detected at early stage of VCI and persists over the course of VCI.^33^, ^34^ BBB consists of endothelial cells, basal lamina matrix, astrocyte end‐feet, and pericytes.^35^ Disruption of BBB can prompt the transfer of inflammatory cells as well as other serum substances such as fibrinogens and complement components into the CNS and then release a variety of cytokines, which in turn activate inflammatory responses and cause WM damage.^36^ Disruption of endothelial tight junctions is responsible for increased BBB permeability and secondary damage to white matter after chronic cerebral hypoperfusion.^10^ Protective effects of NBP on both endothelial cells and BBB have been suggested in a variety of studies. dl‐3‐n‐butylphthalide has been reported to protect endothelial cells against oxidative injury,^37^ reduce cerebral edema, and maintain BBB integrity by inhibiting the protein expression of RhoA in cerebral cortex surrounding rats focal cerebral infarction.^38^

Moreover, activation of MMPs by hypoxia has been demonstrated to be involved in BBB opening via disrupting basal lamina and tight junction proteins.^7^, ^31^ MMP‐2, constitutively expressed in the astrocytic end‐feet close to the cerebral vessels, is activated by hypoxia, which results in disruption of basal lamina and tight junction proteins and subsequent infiltration of inflammatory cytokines.^39^ Then, inducible MMPs, like MMP‐3 and MMP‐9, are activated, which exacerbates BBB damage and inflammatory responses.^40^ In addition, hypoxia‐induced MMPs activation also damages white matter directly by breaking myelinated fibers into myelin basic protein.^7^, ^41^ Studies have shown that down‐regulation of MMP‐9 activity by NBP alleviated brain edema and BBB disruption, illustrated as increased expression of claudin‐5 and decreased BBB permeability in mice with cerebral infarction.^42^ NBP treatment also prevented the increase of MMP‐2 and improved learning and memory ability of rats subjected to permanent bilateral common carotid artery occlusion.^43^ Consistently, our findings also showed that treatment with NBP successfully reduced BBB damage and improved cognitive dysfunction induced by BCAS. Chronic cerebral hypoperfusion induced by BCAS increased expression of MMP‐2 and MMP‐9 at protein level post ‐BCAS. However, treatment with NBP inhibited MMP‐2 and MMP‐9 activity, leading to up‐regulated expression of tight junctions claudin‐5 and ZO‐1, decreased BBB leakage. Therefore, our results indicated that treatment of NBP ameliorated BCAS‐induced BBB permeability through inhibition of MMP activity and alleviation of endothelial tight junctions' disruption in BCAS mice.

Furthermore, accumulating data have demonstrated that inflammation also contributes to chronic cerebral hypoperfusion‐induced white matter lesions.^14^ BBB breakdown interacting with neuroinflammation aggravates cerebral hypoperfusion‐induced white matter damage.^35^ Increased levels of pro‐inflammatory cytokines, including TNFα and IL‐6 in serum and cerebral spinal fluid, were detected in patients with cognitive impairment.^11^, ^44^ In contrast, inhibiting reactive astrogliosis, as well as inactivation of IL‐1β signaling, contributes to amelioration of white matter injury and cognitive deficits.^45^, ^46^, ^47^ In the present study, we found that the number of GFAP‐positive astrocytes was increased in corpus callosum and striatum in BCAS mice, which was significantly suppressed by treatment with NBP. Production of pro‐inflammatory cytokines like TNFα and IL‐6 was also decreased in LPS‐treated astrocytes in vitro by NBP. Consistent with our results, NBP has been demonstrated to inhibit neuroinflammation in several neurological diseases, including ischemic stroke, multiple sclerosis, AD, and vascular dementia.^16^, ^17^, ^22^, ^48^ NBP administration inhibited reactive astrogliosis and ameliorated cognitive deficits in rats with VCI due to chronic cerebral hypoperfusion induced by bilateral common carotid artery occlusion.^22^ Moreover, treatment with NBP also suppressed Aβ‐induced inflammation and production of TNFα and IL‐6 in cultured primary rat astrocytes.^49^ Taken together, these results suggested that NBP inhibited chronic cerebral hypoperfusion‐induced neuroinflammation partially through reducing astrocyte reactivity, and subsequently contributing to improvement in cognitive function of BCAS mice.

The clinical trial of neuroprotection in ischemia has a long history of failure due in part to lack of combination with successful reperfusion. As our results showed, NBP had a therapeutic effect in BCAS mice, which improved CBF. Timely and successful restoration of blood flow may be essential to deliver therapeutic concentrations of neuroprotectants to the ischemic brain.^50^

There are several limitations to the present study. First, other vascular risk factors predisposing white matter dysfunction and VCI, such as hypertension, diabetes mellitus, hypercholesterolemia, aging, and gender, were not reproduced in BCAS mice in this study. Taking vascular risk factors into consideration is worthy for future investigation. Second, although chronic cerebral hypoperfusion induced by BCAS is a well‐established animal model for VCI, it cannot mimic all aspects of pathogenesis of VCI. The CBF falls acutely followed by gradually spontaneous recovery after BCAS. In contrast, cerebral hypoperfusion in patients with VCI often progressed slowly over a longer time. Moreover, it cannot reproduce small vessel diseases, a major pathology for VCI. Recent studies showed that a novel mice model featured with gradual CBF reduction might be a more promising model of VCI. Thus, further studies using this novel murine model of VCI to elucidate the pathogenesis of VCI and potential therapeutic targets of NBP are needed.

In conclusion, our findings in this study suggest the beneficial effects of NBP on white matter integrity and functional recovery through suppressing astrocytes reactivity and BBB breakdown, which at least partially is associated with inhibition of MMPs activity and maintenance of endothelial tight junctions in a murine model of VCI. Treatment with NBP may represent an effective therapeutic strategy for patients with VCI.

## CONFLICT OF INTEREST

The authors declare that they have no conflict of interest.
